# Reconciling bubble nucleation in explosive eruptions with geospeedometers

**DOI:** 10.1038/s41467-020-20541-1

**Published:** 2021-01-12

**Authors:** Sahand Hajimirza, Helge M. Gonnermann, James E. Gardner

**Affiliations:** 1 Department of Earth, Environmental and Planetary Sciences, Rice University, Houston, TX USA; 2 Jackson School of Geosciences, University of Texas at Austin, Austin, TX USA

**Keywords:** Natural hazards, Volcanology

## Abstract

Magma from Plinian volcanic eruptions contains an extraordinarily large numbers of bubbles. Nucleation of those bubbles occurs because pressure decreases as magma rises to the surface. As a consequence, dissolved magmatic volatiles, such as water, become supersaturated and cause bubbles to nucleate. At the same time, diffusion of volatiles into existing bubbles reduces supersaturation, resulting in a dynamical feedback between rates of nucleation due to magma decompression and volatile diffusion. Because nucleation rate increases with supersaturation, bubble number density (BND) provides a proxy record of decompression rate, and hence the intensity of eruption dynamics. Using numerical modeling of bubble nucleation, we reconcile a long-standing discrepancy in decompression rate estimated from BND and independent geospeedometers. We demonstrate that BND provides a record of the time-averaged decompression rate that is consistent with independent geospeedometers, if bubble nucleation is heterogeneous and facilitated by magnetite crystals.

## Abstract (web summary)

The authors simulate bubble nucleation in silica-rich magma with conditions appropriate for Plinian eruptions. They demonstrate that the gap between decompression rate estimates from bubble number density and independent geospeedometers can be largely closed if nucleation is heterogenous facilitated by magnetite crystals and decompression rate is calculated as time-averaged values.

## Introduction

**Fig. 1:**
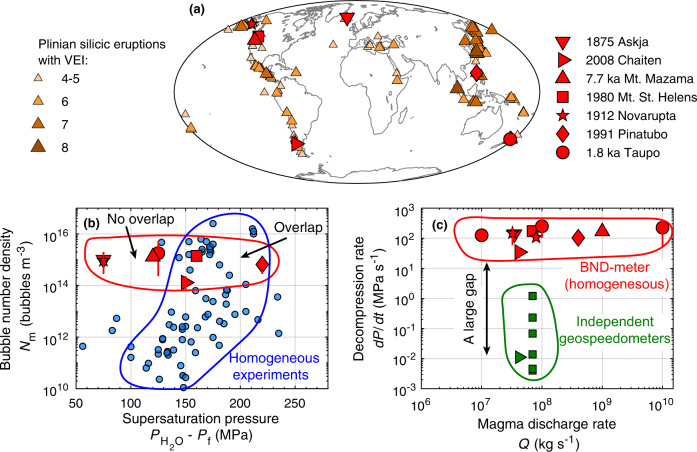
Plinian silicic eruptions, their observed bubble number densities, and their inferred decompression rates. **a** Spatial distribution of Plinian (Volcanic Explosivity Index ≥ 4) silicic eruptions over the past 100 kyr, based on Crosweller et al.^2^. Red symbols are eruptions for which bubble number density and H_2_O saturation pressure are documented. They are: 1875 Askja^40,41^; 2008 Chaiten^42,43^; 7.7 ka Mount Mazama^44,45^; 1980 Mount St. Helens (MSH)^46–48^; 1912 Novarupta^49,50^; 1991 Pinatubo^51,52^; 1.8 ka Taupo^53^ eruptions. **b** Bubble number density versus the maximum potential H_2_O supersaturation pressure for eruptions (red symbols) and for homogeneous nucleation experiments (blue symbols)^10,12,19,32,54–59^. Only experiments with supersaturation pressure of ≥150 MPa overlap with eruptions. **c** Decompression rate values estimated from observed bubble number density and homogeneous nucleation^17^. There is a large gap between these estimates and those calculated by independent geospeedometers for the same eruptions^43,60–64^.

Plinian eruptions are among Earth’s most explosive volcanic events and are typically associated with magmas of high-silica content^1^. In the past 100 kyr over 500 Plinian silicic eruptions with Volcanic Explosivity Index of four or greater have occurred globally^2^ (Fig. 1a). These eruptions have considerable destructive power and present extensive risks, both locally (e.g., pyroclastic density current) and globally (e.g., atmospheric ash and aerosol dispersal). The destructive potential of such eruptions derives from numerous bubbles that nucleate and grow during magma ascent^3,4^. These bubbles contain a highly compressible fluid mixture of exsolved magmatic volatiles, predominantly H_2_O ^5^. Bubble overpressure, relative to the surrounding melt, provides the potential energy for magma fragmentation, which results in explosive eruptions^6^. The development of overpressure in bubbles is thought to depend on the rate at which magma decompresses during ascent^7,8^. Because eruptive processes are inaccessible to direct observation, understanding explosive volcanism is contingent upon reconstructing governing processes and their controlling parameters from indirect observations. The number density of bubbles, preserved in erupted pyroclasts, is such an independent observation and of critical importance to elucidating the dynamical feedback between magma decompression, H_2_O exsolution, and explosive magma fragmentation.

Bubble nucleation rate and the resultant bubble number density are governed by the feedback between H_2_O exsolution and magma decompression^4^. The latter is a consequence of the combined decrease in static pressure and pressure loss from viscous resistance to flow as magma rises toward the surface^6^. Consequently, decompression rate depends dynamically on magma discharge rate, conduit dimensions, and magma viscosity, which increases as H_2_O exsolves into bubbles by diffusion^9^. The efficiency of diffusion, in turn, is rate limited by the number density of bubbles, such that slow diffusion kinetics facilitates large supersaturations and high rates of bubble nucleation^4^. Bubble number density provides a record of this feedback. Reconstruction of these processes and reliable estimation of magma decompression rate require quantitative models of bubble nucleation that are calibrated with experiments.

Bubble number densities preserved in pyroclasts from Plinian silicic eruptions span a narrow range of 10^15 ± 1^ m^−3^ despite more than 3 wt% variation in pre-eruptive H_2_O concentrations (Fig. 1b). Figure 1b compares the observed bubble number densities with experimental results of homogeneous nucleation. The data are presented in terms of the potential maximum supersaturation pressure. In case of experiments, it is the difference between the H_2_O saturation pressure and the final pressure, whereas for eruptions, it is the difference between saturation pressure and atmospheric pressure. Homogeneous nucleation typically initiates at supersaturation pressures of ≈110 MPa^10^. Bubble number density increases with supersaturation pressure and reaches the range of bubble number density observed in pyroclasts at supersaturation pressures of >150 MPa. Such high pressures, however, are greater than the potential maximum supersaturation pressure for most eruptions^11^. Moreover, the conventional estimates of decompression rate based on homogeneous nucleation are unrealistically high (~100 MPa s^−1^), do not correlate with magma discharge rate, and are substantially greater than estimates from independent geospeedometers, which are ≤1 MPa s^−1^ (Fig. 1c). To resolve these discrepancies, Shea^11^ hypothesized that bubble nucleation is perhaps heterogeneous and facilitated by abundant pre-existing crystals. The heterogeneous nucleation hypothesis, however, does not close the gap between decompression rate estimates from bubble number density and independent geospeedometers.

The purpose of this study is to reconcile observed bubble number densities in Plinian silicic eruptions with independent geospeedometers. We integrate Hajimirza et al.’s^12^ bubble nucleation model, obtained by calibrating classical nucleation theory against experiments, with a model of magma flow in the conduit during Plinian eruptions^4,13,14^. We find that the vast majority of eruptions require heterogeneous nucleation, perhaps due to the presence of magnetite crystals. This is consistent with Shea’s hypothesis^11^. We assess the implications of heterogeneous bubble nucleation on the conduit flow, with a focus on magma decompression rates. Conduit flow models indicate that the decompression rate during magma ascent is not constant. Rather it increases as H_2_O exsolves and viscosity increases^6,15^. Our model simulations account for this time-dependent magma decompression and are more consistent with the fluid dynamics of magma ascent. This allows for the estimation of a time-averaged decompression rate, defined as the ratio of total decompression over total ascent time. The time-averaged decompression rate is well suited for comparison with independent geospeedometers, which yield decompression rate estimates within close range of the time-averaged values^16^. In contrast, estimates based on scaling relations, such as BND meter of Toramaru^17^ which is widely used^11^, yields values that are more representative of decompression rates at the peak of nucleation^11^ and can be orders of magnitude higher than time-averaged estimates. By accounting for the aforementioned time-dependent feedback during magma decompression, we find that the time-averaged decompression rates estimated from BND under heterogeneous nucleation are consistent with independent geospeedometers.

## Results

### Bubble nucleation

Bubble nucleation is the formation of molecular clusters that are larger than a critical size and stable to grow into bubbles. Nucleation is driven by thermodynamic disequilibrium due to supersaturation of dissolved volatiles, as magma decompresses to a pressure below its saturation pressure. The change in free energy, *W*, associated with the formation of bubble nuclei is quantified by classical nucleation theory^18^. *W* derives from the balance between a reduction in free energy, caused by the clustering of volatile molecules, and an increase in free energy, caused by the formation of a new interface between molecules within the cluster and the surrounding silicate. The bubble nucleation rate depends exponentially on *W*^18^. Bubble nuclei are of the order of a few nanometers in size^12,18^ and will grow into micron- to millimeter-size bubbles by diffusion, which tends to reduce supersaturation.

We simulate bubble nucleation and growth during magma decompression in order to examine the conditions under which bubbles in Plinian pyroclasts may have nucleated (see “Methods” section for details). We consider H_2_O as the dominant volatile phase because it is the most abundant^5^ and primarily controls the final bubble number density^4^. Our simulations predict nucleation rate during decompression from an initial saturation pressure until magma fragmentation. Decompression rate is estimated for steady flow of magma within a cylindrical conduit of constant cross-sectional area using the Darcy–Weisbach relation^6^. Dependent parameters are: pressure in the surrounding melt, average concentration of dissolved H_2_O in the melt, nucleation rate, bubble number density, average bubble size, pressure inside bubbles, and bubble volume fraction. We use the nucleation model of Hajimirza et al.^12^, which has been calibrated against homogeneous bubble nucleation experiments in rhyolite, and reliably predicts experimental results over a wide range of saturation pressures and decompression rates.

The homogeneous nucleation energy, *W*_Hom_, is large and a high supersaturation pressure is required to overcome the surface energy barrier for nucleus formation^10,12^. In our simulations, we first examined whether the observed bubble number density in each eruption can be produced by homogeneous nucleation. We find that the 1991 eruption of Pinatubo is the only eruption where the observed bubble number density can be attained by homogeneous nucleation. For all other eruptions a necessary reduction in nucleation energy, relative to the homogeneous value, is required. This reduction could be attained by the presence of dissolved fluorine. For example, 1 wt% fluorine reduces nucleation energy by 75%^19^. Typical fluorine concentrations in explosively erupted magmas, however, are only ~200–1500 ppm^5^. An alternative is heterogeneous nucleation on crystals^18^, including nanometer-size nanolites^11^. Here we assess the effect of heterogeneous nucleation on magma ascent dynamics during Plinian eruptions.

### Reconciling bubble nucleation with eruption dynamics

**Fig. 2:**
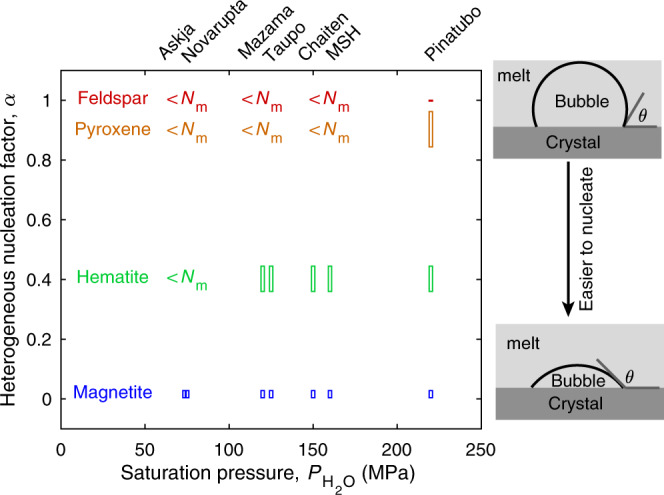
Heterogeneous nucleation on magnetite reconciles H_2_O saturation pressure with bubble number densities. Heterogeneous nucleation factor, *α*, required for each eruption to match bubble number densities. Pinatubo can be reconciled with homogeneous nucleation (*α* = 1), whereas all other eruptions require heterogeneous nucleation (*α* < 1). *α* shown are for magnetite^20,21^, hematite^21,23^, pyroxene^22^, and feldspar^20^. Magnetite is the only mineral phase that allows heterogeneous nucleation to simultaneously match observed bubble number densities in all eruptions.

Heterogeneous nucleation in magmatic systems is facilitated by the presence of crystalline molecular aggregates that provide nucleation sites for bubbles. Heterogeneous nucleation facilitates nucleation by reducing the homogeneous nucleation energy by a factor, 0 < *α* < 1. The value of *α* during such heterogeneous nucleation is described as a function of the dihedral contact angle, *θ*, between the melt-bubble interface and the pre-existing crystal (Fig. 2). Direct measurements of *θ* for bubble nuclei are impossible because nuclei are too small and ephemeral. Some studies have attempted to estimate *θ* from the contact angle between microscopically observed bubbles and crystals^20^. It is, however, unlikely that the contact angle is the same for nuclei and microscopically observable bubbles because their thermodynamic properties are different^12,18^. Instead, *θ* has been inferred from the difference in pressure, Δ*P*, at which bubbles first nucleate during decompression in homogeneous and heterogeneous nucleation experiments, with $\alpha \,=\,{({{\Delta }}{P}_{{\rm{Het}}}/{{\Delta }}{P}_{{\rm{Hom}}})}^{2}$^18,21^. Based on such experiments it has been shown that the contact angle is dependent on the substrate’s mineralogical structure (Fig. 2). For example, the contact angle for feldspar is ~0–20°^20^, for pyroxene is 40–60°^22^, whereas for hematite is ~90–100°^21,23^, and for magnetite is ~145–160°^20,21^. The contact angles for feldspar and pyroxene are too low to allow heterogeneous nucleation to match the observed bubble number densities (Fig. 2). Nucleation on hematite can match observed bubble number densities in most eruptions. To the best of our knowledge, however, hematite does not occur naturally in most magmas. Magnetite is the most effective mineral phase for heterogeneous bubble nucleation and is the only mineral phase that can cause heterogeneous nucleation to produce bubbles in sufficient numbers in all eruptions considered.

**Fig. 3:**
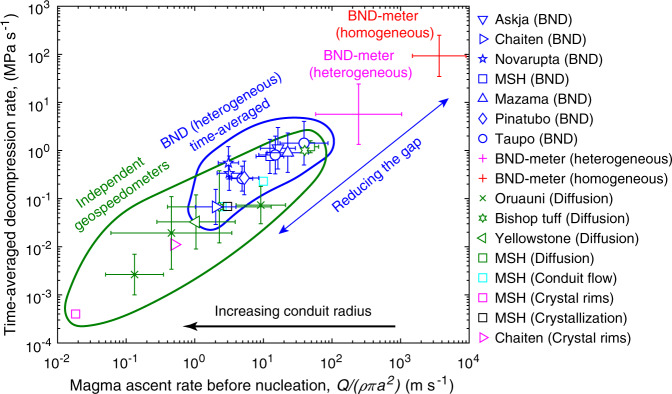
Estimated decompression rates for silicic Plinian eruptions. The blue symbols show the time-averaged estimates from bubble number density with heterogeneous nucleation on magnetite. The error bars represent uncertainties in magnetite contact angle. Heterogeneous nucleation substantially reduces the gap in decompression rate between homogeneous nucleation and independent geospeedometers that include: diffusion in melt inclusions and melt embayments for May 18th, 1980 Mt. St. Helens^60^, 0.77 Ma Bishop tuff^65,66^, 27 ka Oruauni^66–68^, and 2 Ma Yellowstone^66^; conduit models for May 18th, 1980 Mt. St. Helens^61,62^; crystal rims for May 18th, 1980 Mt. St. Helens^63^ and 2008 Chaiten^43^; and groundmass crystallization for May 18th, 1980 Mt. St. Helens^64^.

For each eruption we ran simulations wherein homogeneous nucleation energy is scaled with *α* for the range of possible contact angles for magnetite (145–160°). The simulation parameters are given in Supplementary Table 1. The time-averaged decompression rates, at which the magma would be predicted to fragment with a bubble number density equal to each eruption, range between 0.1 and 1 MPa/s (Fig. 3). These estimates reconcile observed bubble number densities with nucleation theory and the fundamental fluid dynamics of magma ascent^6^. The time-averaged decompression rates are more than one order of magnitude lower than equivalent values predicted by the BND meter for heterogeneous nucleation^17^. The reason for this discrepancy is because decompression rates from the BND meter are representative of peak rates. This is an important distinction because independent geospeedometers, which tend to be based on diffusion kinetics, provide estimates that closely approximate time-averaged values^16^. Thus, the time-averaged decompression rates obtained from our simulations largely eliminate the gap with independent geospeedometers (Fig. 3). Despite heterogeneous nucleation generating sufficient numbers of bubbles at more realistic slower decompression rates, those rates still exceed geospeedometer estimates, which we speculate to be indicative of a widening conduit with depth.

## Discussion

Our simulation results suggest that bubble number densities can be reconciled with pre-eruptive H_2_O concentration and independent geospeedometers, if nucleation is heterogeneous due to magnetite. For none of the eruptions magnetite crystals have been reported at number densities similar to or greater than bubble number densities. This does not, however, rule out the presence of magnetite because they are typically much smaller than bubbles and might be undercounted in 2D scanning electron microscopy (SEM) images^11,24^. Furthermore, magnetite crystals might exist at sizes well below the resolution of SEM images. For example, magnetite nanolites as small as 20 nm are reported in samples from Paintbrush Tuff (USA)^25^, and are discovered in samples from Green Tuff (Italy) and Yellowstone (USA) using Raman spectroscopy^26,27^. Using transmission electron microscopy, Mujin et al.^24^ observed magnetite nanolites in samples from Shinmoedake Volcano (Japan) with sizes down to 1 nm and number densities of up to ~10^23^ m^−3^.

Our analysis is based on the hypothesis that heterogeneous nucleation sites exist during eruptive magma ascent. This is supported by the fact that bubble nucleation experiments require extensive treatment at superliquidus conditions in order to avoid heterogeneous nucleation, in some cases without observable crystals^20,28^. The hypothesized existence of abundant oxides in erupting magmas at subliquidus conditions is thus not unreasonable^11^. By the same token, homogeneous bubble nucleation experiments, while necessary as a basis for understanding nucleation, may not fully encapsulate bubble formation in volcanic eruptions.

**Fig. 4:**
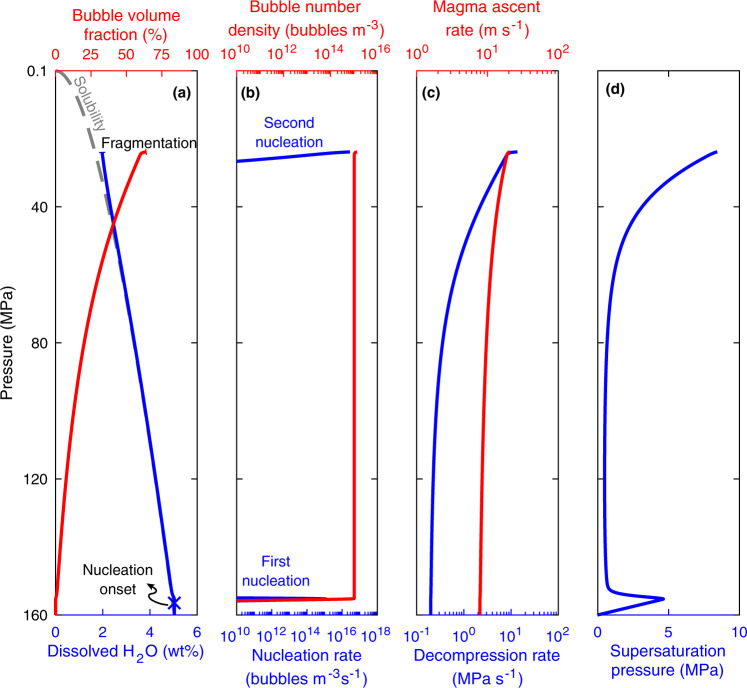
Illustrative model results of the feedback between water exsolution, decompression rate, and magma fragmentation during heterogeneous nucleation. The contact angle is *θ* = 160° (*α* = 0.003). Nucleation first occurs at low supersaturation. Subsequently H_2_O concentration remains close to equilibrium because of H_2_O diffusion into nucleated bubbles. This results in a progressive increase in viscosity and hence decompression rate. Supersaturation pressure increases gradually, leading to a second nucleation peak, followed by magma fragmentation.

Heterogeneous nucleation exerts a complex feedback between H_2_O exsolution, decompression rate, and explosive magma fragmentation. Figure 4 provides a representative example of model results. Heterogeneous nucleation starts shortly after magma decompression. After the onset of nucleation H_2_O diffuses into the existing bubbles. Bubbles grow, resulting in progressive decrease of the characteristic diffusion length, which enhances the diffusion of H_2_O into bubbles. The average dissolved H_2_O concentration therefore remains close to equilibrium as the magma continues to decompress. The initial decompression rate is predominantly due to the reduction in hydrostatic pressure, which is proportional to magma ascent rate. As more H_2_O exsolves magma viscosity increases resulting in a continuous increase in decompression rate. Consequently supersaturation gradually increases, resulting in a second nucleation peak of higher rate than the first one. Because of the substantial overpressure in the newly nucleated bubbles, fragmentation conditions are reached immediately after the second nucleation peak. Our simulations predict that under heterogeneous nucleation a second nucleation peak occurs for all eruptions, independent of their saturation pressure. The detailed simulation results for all eruptions are given in Supplementary Table 2.

In summary, we find that bubbles at number densities in pyroclastic samples from a wide range of Plinian silicic eruptions are consistent with heterogeneous nucleation due to magnetite, if present at number densities similar to those discovered in several explosive eruptions^24–27^. Such heterogeneous bubble nucleation can resolve the discrepancy between the inferred water saturation for many eruptions and that required to nucleate bubbles. By accounting for the time-varying decompression rate, arising due to feedbacks between H_2_O exsolution and magma viscosity, we overcome the peak decompression rate bias of conventional methods. We thus calculate time-averaged decompression rates that can be compared to independent geospeedometers. We find that heterogeneous nucleation largely closes the gap with independent geospeedometers. Heterogeneous bubble nucleation due to magnetite is a viable hypothesis that provides impetus for future investigations, in particular a systematic search for the presence of magnetite nanolites in Plinian samples.

## Methods

### Model assumptions

We simulated bubble nucleation and growth during magma ascent in a vertical cylindrical conduit with a constant cross-sectional area. For a given eruption, simulations commence from H_2_O saturation pressure and stop when fragmentation conditions are met.

The assumption for magma flow in the conduit are: steady state because the time scale for Plinian eruptions is substantially longer than the time scale of magma ascent in the conduit^29^; one-dimensional flow with flow properties averaged over the cross-sectional area of the conduit^6^; the relative velocity between bubbles and melt is neglected because the drag force associated with silicic melt prevents bubbles from rising independently through the melt^15^; isothermal conditions and nucleation is only driven by decompression^14^; nanolites do not affect magma rheology; nucleation after fragmentation is assumed to be negligible^14^.

The assumptions for bubble nucleation and growth are: magma is initially bubble free; H_2_O exsolution is non-equilibrium; bubble nucleation rate is estimated using classical nucleation theory; nucleation is heterogeneous on abundant pre-existing magnetite crystals (no crystal nucleation); we use the method of moments to estimate the growth rate of bubbles with different sizes^4^; we assume bubble growth is steady state^4^. This is justified because the inertia terms in the diffusion equation are negligible at low supersaturation pressures during heterogeneous nucleation^30^.

### Magma flow in the conduit

Considering the above assumptions, conservation of mass and momentum simplifies to

1 $$\frac{\partial (\rho U)}{\partial z}\,=\,0,$$

and

2 $$\rho U\frac{\partial U}{\partial z}\,=\,-\frac{\partial {P}_{{\rm{m}}}}{\partial z}\,-\,\rho g\,-\,{F}_{{\rm{fric}}},$$

respectively. Here *ρ* is magma density, averaged over melt and gas phases,

3 $$\rho \,=\,\phi {\rho }_{{\rm{g}}}\,+\,(1\,-\,\phi ){\rho }_{{\rm{m}}}.$$

*ϕ* is the volume fraction of bubbles, *ρ*_g_ and *ρ*_m_ are gas and melt densities, respectively, *U* is magma ascent rate, *g* is the gravitational acceleration, and *F*_fric_ is the pressure loss due to friction forces. The latter is calculated from the Darcy–Weisbach relation, *F*_fric_ = 8 *μU*/*a*^2^. *a* is the conduit radius and *μ* is the magma viscosity, given by ${\mu }_{{\rm{m}}}{(1\,-\,{\phi }_{{\rm{crystal}}}/0.6)}^{(-5/2)}$. Here *μ*_m_ is the melt viscosity and *ϕ*_crystal_ is the volume fraction of microlites. Substituting Eq. (1) into Eq. (2) and replacing *U* with *Q*/(*ρπa*^2^) gives the pressure loss as

4 $$\frac{\partial {P}_{{\rm{m}}}}{\partial z}\,=\,-\left(\rho g+\frac{Q}{\rho \pi {a}^{2}}\left(\frac{8\mu }{{a}^{2}}\,-\,\frac{Q}{\rho \pi {a}^{2}}\frac{\partial \rho }{\partial z}\right)\right),$$

where *Q* is the mass discharge rate.

### Bubble nucleation

In our model, we allow for non-equilibrium exsolution, that is H_2_O may become supersaturated as magma decompresses, driving bubble nucleation and growth. We used classical nucleation theory to estimate nucleation rate of stable bubble nuclei at a given supersaturation pressure. Molecular clusters of volatiles are stable and grow into bubbles if they are larger than the critical nucleus size, *R*_c_, given by^18^

5 $${R}_{{\rm{c}}}\,=\,\frac{2\gamma }{{P}_{{\rm{n}}}\,-\,{P}_{{\rm{m}}}},$$

where *γ* is the surface tension of bubble nuclei, *P*_n_ is the pressure inside a bubble nucleus, and *P*_m_ is pressure in the surrounding melt. *P*_n_ is related to the saturation pressure of volatiles, *P*_sat_, through^23^

6 $$f({P}_{{\rm{n}}},T){P}_{{\rm{n}}}\,=\,f({P}_{{\rm{sat}}},T){P}_{{\rm{sat}}}{e}^{{{\Omega }}({P}_{{\rm{m}}}\,-\,{P}_{{\rm{sat}}})/{k}_{{\rm{B}}}T},$$

where *T* is temperature, *f*(*P*, *T*) is the fugacity coefficient of the supersaturated volatile phase, Ω is the volume of volatile molecules, and *k*_B_ is the Boltzmann constant. The homogeneous nucleation energy, *W*_Hom_, is estimated from

7 $${W}_{{\rm{Hom}}}\,=\,\frac{16\pi {\gamma }^{3}}{3{({P}_{{\rm{n}}}\,-\,{P}_{{\rm{m}}})}^{2}},$$

and the nucleation rate is^18,31^

8 $$J\,=\,{J}_{0}\exp \left(-\frac{{W}_{{\rm{Hom}}}}{{k}_{{\rm{B}}}T}\alpha \right),$$

with

9 $${J}_{0}\,=\,\frac{2{{\Omega }}{n}_{0}^{2}D}{{a}_{0}}\sqrt{\frac{\gamma }{{k}_{{\rm{B}}}T}}.$$

*n*_0_ is the concentration of volatiles molecules in the melt, *D* is the diffusion coefficient, *a*_0_ is the average distance between volatiles molecules and *α* is the heterogenous nucleation factor, which depends on the contact angle, *θ*, between bubble nuclei and crystals as

10 $$\alpha \,=\,\frac{(2\,\,-\cos \theta ){(1\,+\,\cos \theta )}^{2}}{4}.$$

The nucleation rate is strongly controlled by surface tension, *γ*, such that a few percent variations in *γ* can change *J* by >10 orders of magnitude^32^. A reliable prediction of nucleation rate, and consequently bubble number density, thus requires a firm constraint on surface tension. Here we use the surface tension formulation defined by Hajimirza et al.^12^, which has been shown to reliably predict observed bubble number density in homogeneous nucleation experiments . *γ* is given by

11 $$\gamma \,=\,\frac{0.49\,{\gamma }_{{\rm{B}}}}{1\,+\,2\delta /{R}_{{\rm{c}}}},$$

where *γ*_B_ is the surface tension measurements for macroscopic bubbles^33^, and *δ* ≈ 0.32 nm is the Tolman length for bubble nuclei in rhyolite^12,34^.

### Bubble growth

When a bubble nucleus forms, the H_2_O concentration at the bubble-melt interface is determined by the solubility of H_2_O at the pressure inside the bubble. This concentration is lower than the concentration in the surrounding melt, resulting in a concentration gradient which drives diffusion of H_2_O molecules toward bubble nuclei. The resultant mass flux of H_2_O into a bubble, *q*, is approximated as,

12 $$q\,=\,D{\left(\frac{\partial c}{\partial r}\right)}_{r \,=\, R}.$$

Here *D* is diffusion coefficient, *r* is the distance from bubble’s center, *R* is bubble radius, and *c* is the water concentration in the surrounding melt, given by^7^

13 $$\frac{\partial c}{\partial t}\,+\,\frac{dR}{dt}\frac{\partial c}{\partial z}\,=\,\frac{1}{{r}^{2}}\frac{\partial c}{\partial r}\left(D{r}^{2}\frac{\partial c}{\partial r}\right).$$

Chernov et al.^30^ demonstrated that at low supersaturation pressures the inertial terms, the left-hand side in Eq. (13), are negligible. We thus neglect those terms because in heterogeneous nucleation supersaturation pressure remains low. With boundary conditions *c*(*r* = *R*) = *C*_R_ and *c*(*r* → *∞*) = *C*_m_, where *C*_m_ and *C*_R_ are the average H_2_O concentrations in the melt and at the bubble-melt interface, respectively, *c* is estimated as^30^

14 $$c\,=\,{C}_{{\rm{m}}}\,-\,({C}_{{\rm{m}}}\,-\,{C}_{{\rm{R}}})\frac{R}{r}.$$

The mass of H_2_O inside the bubble, *m*_b_, will increase due to diffusion at a rate

15 $$\frac{d{m}_{{\rm{b}}}}{dt}\,=\,4\pi {R}^{2}{\rho }_{{\rm{m}}}q.$$

The bubble growth rate is given by

16 $$\frac{dR}{dt}\,=\,\frac{R}{4\mu }\left({P}_{{\rm{b}}}\,-\,{P}_{{\rm{m}}}\,-\,\frac{2\gamma }{R}\right),$$

which accounts for bubble growth driven by diffusion as well as decompression. Here *μ* is the viscosity of melt surrounding the bubble, *P*_b_ is the pressure inside the bubble, estimated using the equation of state of H_2_O, and *d*/*dt* = *U*∂/∂*z* is the material derivative of a given quantity in steady state. Inertial terms in Eq. (16) are neglected, given that they are considerably smaller than the viscous terms^4^.

The above equations describe the growth rate of a single bubble. Because the number of bubbles in the magma is too high to track growth rates for each individual bubble, we use the method of moments, which calculates the moments of size distributions. The *k*th moment is defined as^4^

17 $${M}_{k}(t)\,=\,\mathop{\int}\nolimits_{0}^{\infty }{R}^{k}{{\Lambda }}\left(R, t\right)\ dR,$$

where Λ is number of bubbles with radii in the interval of *R* and *R* + *dR* per unit volume of melt. Each moment refers to a measurable characteristic quantity^4^: *M*_0_ is the total number of bubbles per unit volume of melt (BND), *M*_1_ is the total radius of bubbles per unit volume of melt, 4*πM*_2_ is the total surface area of bubbles per unit volume of melt, and $\frac{4\pi }{3}{M}_{3}$ is the total volume of bubbles per unit volume of melt. The volume fraction of bubbles in Eq. (3) is estimated from $(\frac{4\pi }{3}{M}_{3})/(1\,+\,\frac{4\pi }{3}{M}_{3})$.

The evolution of bubble size distribution due to the growth of existing bubbles and nucleation of new bubbles is given by the population balance equation as^13^

18 $$\frac{d{{\Lambda }}(R,t)}{dt}\,+\,G(\hat{R})\frac{\partial \left({{\Lambda }}(R,t)\right)}{\partial R}\,=\,J\mathop{\int}\nolimits_{0}^{\infty }\delta (R\,-\,{R}_{{\rm{c}}})\ dR.$$

*δ* is the Dirac delta function and $G(\hat{R})$ is the growth rate of bubbles, assumed to be equal for all bubbles and estimated from Eq. (16) for a bubble of mean radius, $\hat{R}\,=\,{M}_{1}/{M}_{0}$. The evolution of the zeroth moment through time is given by

19 $$\frac{d{M}_{0}}{dt}\,=\,J,$$

and the evolution of the higher-order moments, *k* ≥ 1, is given by

20 $$\frac{d{M}_{k}}{dt}\,=\,kG(\hat{R}){M}_{k\,-\,1}\,+\,J{R}_{{\rm{c}}}^{k}.$$

The concentration of H_2_O dissolved within the melt decreases as a result of the diffusion of water into bubbles. Mass conservation of H_2_O requires that

21 $$\frac{d{C}_{{\rm{m}}}}{dt}\,=\,-\frac{1}{{\rho }_{m}}\left({M}_{0}\frac{d{m}_{{\rm{b}}}}{dt}\,+\,J{m}_{{\rm{c}}}\right),$$

where *ρ*_m_ is the melt density, assumed to be constant throughout magma decompression, and *m*_c_ is the mass of a bubble nuclei estimated from the equation of state.

### Model simulation

For each eruption, we integrated Eqs. (4, 19, and 20) for *k* = 1 through 3, as well as Eq. (21), using the ode15s function of MATLAB^®^. Each simulation initiated at the known saturation pressure and with additional initial conditions of

22 $${M}_{k}\,=\,0,\quad {P}_{{\rm{m}}}\,=\,{P}_{{{\rm{H}}}_{2}{\rm{O}}}\quad {\rm{and}}\quad {C}_{{\rm{m}}}\,=\,{C}_{{{\rm{H}}}_{2}{\rm{O}}},$$

where ${C}_{{{\rm{H}}}_{2}{\rm{O}}}$ is related to ${P}_{{{\rm{H}}}_{2}{\rm{O}}}$ through the H_2_O solubility relation^35^. A given simulation ends when the fragmentation criterion of Papale^36^ is reached. The values of input variables for each eruption are given in Supplementary Table 1.

The objective of our model simulation is to estimate decompression rate, conditional on the observational constraints of measured bubble number density and magma fragmentating. All parameters in the governing system of equations are either specified or calculated from existing formulations: H_2_O solubility^35^, diffusion coefficient^37^, equation of state^38^, fugacity coefficient^38^, surface tension^12^, melt viscosity^9^, and the molecular volume of H_2_O^39^. Conduit radius, which is related to decompression rate through equation (4), is the only parameter that is not constrained. For each eruption, the model simulations predict a conduit radius and an average decompression rate that are conditional on the aforementioned observational constraints. The values of output variables for each eruption are given in Supplementary Table 2.

## Supplementary information

Supplementary Information

## Data Availability

The authors declare that the data that support the findings of this study are available within the paper and the supplementary tables.

## Code availability

All equations used in the code are presented in the “Methods” section. The code is available at "https://github.com/Sahand-Hajimirza/BND-speedometer".
