# Supplementary material for: Reconciling bubble nucleation in explosive eruptions with geospeedometers
Source: Nat Commun. 2021 Jan 12;12:283. doi: 10.1038/s41467-020-20541-1 (PMC7803785; doi:10.1038/s41467-020-20541-1)
Supplement: Supplementary file 1 — Supplementary Information [file 41467_2020_20541_MOESM1_ESM.pdf]

Supplementary information

Reconciling bubble nucleation in explosive eruptions with geospeedometers

Sahand Hajimirza,[1,a] Helge M. Gonnermann,[1] and James E. Gardner[2]

[1] *Department of Earth, Environmental and Planetary Sciences, Rice University, Houston, TX, USA*

[2] *Jackson School of Geosciences, University of Texas at Austin, Austin, TX, USA*

[a] Electronic mail: sahand@rice.edu

Supplementary information

Supplementary Table I. Simulation parameters. The references for the data are provided in the main text.

Eruption (phase)	H_2O saturation pressure (MPa)	Temperature ($°C$)	Crystal volume fraction	Mass discharge rate (10^7 kg s^{-1})	Bubble number density (10^{15} m^{-3})
1875 Askja (D)	75	1000	0	3	1.0
2008 Chaiten (May 6th)	150	800	0	4	0.1
7.7 ka Mt. Mazama	120	880	0	100	1.3
1980 Mt. St. Helens (May 18th)	160	900	0.3	7	1.4
1912 Novarupta (2)	75	830	0.4	8	0.7
1912 Novarupta (3)	75	830	0.4	3	1.1
1991 Pinatubo (C)	220	780	0.47	40	0.6
1.8 ka Taupo (2)	125	860	0	1	0.9
1.8 ka Taupo (5)	125	860	0	10	2.4
1.8 ka Taupo (6)	125	860	0	1000	2.1

Supplementary Table II. The range of simulation results for heterogeneous nucleation on magnetite.

Eruption (phase)	Pressure at first nucleation (MPa)	Pressure at second nucleation (MPa)	Bubble volume fraction	Conduit radius (m)
1875 Askja (D)	61-70	13-10	0.70-0.77	13-23
2008 Chaiten (May 6th)	139-146	11-8	0.79-0.83	38-75
7.7 ka Mt. Mazama	108-116	15-8	0.68-0.78	55-110
1980 Mt. St. Helens (May 18th)	149-156	44-31	0.60-0.64	22-35
1912 Novarupta (2)	62-71	16-18	0.56-0.57	48-72
1912 Novarupta (3)	62-71	19-23	0.52-0.50	32-45
1991 Pinatubo (C)	210-216	69-43	0.45-0.54	80-130
1.8 ka Taupo (2)	114-121	23-21	0.78-0.70	14-20
1.8 ka Taupo (5)	114-121	28-18	0.62-0.69	23-40
1.8 ka Taupo (6)	114-121	17-6	0.64-0.80	125-280
